# The Maestro (*Mro*) Gene Is Dispensable for Normal Sexual Development and Fertility in Mice

**DOI:** 10.1371/journal.pone.0004091

**Published:** 2008-12-31

**Authors:** Lee Smith, John Willan, Nick Warr, Frances A. Brook, Michael Cheeseman, Richard Sharpe, Pam Siggers, Andy Greenfield

**Affiliations:** 1 Mammalian Genetics Unit, MRC Harwell, Oxfordshire, United Kingdom; 2 Department of Zoology, University of Oxford, Oxford, United Kingdom; 3 MRC Human Reproductive Sciences Unit, The Queen's Medical Research Institute, Edinburgh, United Kingdom; Temasek Life Sciences Laboratory, Singapore

## Abstract

The mammalian gonad arises as a bipotential primordium from which a testis or ovary develops depending on the chromosomal sex of the individual. We have previously used DNA microarrays to screen for novel genes controlling the developmental fate of the indifferent embryonic mouse gonad. Maestro (*Mro*), which encodes a HEAT-repeat protein, was originally identified as a gene exhibiting sexually dimorphic expression during mouse gonad development. Wholemount *in situ* hybridisation analysis revealed *Mro* to be expressed in the embryonic male gonad from approximately 11.5 days *post coitum*, prior to overt sexual differentiation. No significant expression was detected in female gonads at the same developmental stage. In order to address its physiological function, we have generated mice lacking Maestro using gene targeting. Male and female mice homozygous for a *Mro* null allele are viable and fertile. We examined gonad development in homozygous male embryos in detail and observed no differences when compared to wild-type controls. Immunohistochemical analysis of homozygous mutant testes of adult mice revealed no overt abnormalities. Expression profiling using DNA microarrays also indicated no significant differences between homozygote embryonic male gonads and controls. We conclude that Maestro is dispensable for normal male sexual development and fertility in laboratory mice; however, the *Mro* locus itself does have utility as a site for insertion of transgenes for future studies in the fields of sexual development and Sertoli cell function.

## Introduction

The differentiation of the mammalian gonad from an indifferent embryonic primordium is controlled by distinct molecular mechanisms in males and females. In males, the expression of the Y-linked testis determining gene, *SRY*, initiates a pathway of genetic and cellular activity that culminates in the differentiation of the testis [1]–[4]. In females, the absence of *SRY* results in ovary development. Whilst progress in understanding the molecular basis of ovary development has been slower, several genes have now been demonstrated to function in the pathway of testis development, many of these comprising transcription factors or secreted signalling molecules expressed in a male-specific fashion during gonad development (reviewed in [5], [6]). In order to contribute novel genes to our understanding of these pathways of sexual development we used DNA microarrays to screen for transcripts exhibiting sexually dimorphic patterns of expression during mouse gonadogenesis [7], [8].

One gene identified by such a screen was Maestro (*Mro*) [9]. *Mro* expression was examined by wholemount in *situ* hybridisation (WMISH) analysis of the developing male and female gonads and was detected only in the developing male gonad. *Mro* expression was first observed at approximately 11.5 days *post coitum* (dpc) in the undifferentiated male gonad. By 13.5 dpc *Mro* transcripts were restricted to the developing testis cords and appeared to be found in both the somatic (Sertoli) cells and germ cells of the cords. This sexually dimorphic expression of *Mro* has been observed in two independent studies of mouse gonad development using quantitative RT-PCR [10] and DNA microarrays [11]. Extra-gonadal sites of expression in adult mice were determined by Northern analysis and included the heart, brain, and liver [9].

*Mro* encodes a predicted protein of 248 amino acids that comprises four HEAT repeat motifs. HEAT repeats were first identified in a functionally diverse group of proteins including huntingtin, elongation factor 3, the PR65/A subunit of protein phosphatase 2A (PP2A) and the lipid kinase TOR1 [12], and are thought to mediate protein-protein interactions. Transfection of Maestro-GFP fusion constructs into different mammalian cell lines reveals a predominantly nucleolar localisation for the protein [9]. Along with the conventional role of the nucleolus in ribosome biogenesis, additional roles in the modulation of diverse molecular pathways have been described recently [13], [14].

The expression pattern of Maestro during embryonic gonadogenesis suggests a possible function in testis development. Moreover, the nature of its encoded protein and subcellular localisation promise novel insights into the molecular and cellular basis of gonad development in mammals. In order to define its potential role in mouse sexual development we generated mice lacking Maestro using gene targeting. Here, we report the characterisation of sexual development in male mutant homozygotes and a more general examination of the mutant phenotype.

## Materials and Methods

### Gene targeting and mouse breeding

A Maestro (*Mro*) genomic clone was isolated from a 129SvJ BAC library (CITB-CJ7-B, Research Genetics) as previously described [9]. The *Mro* targeting construct was generated in pBluescript II SK+ (Stratagene). The 5′ homology arm consisted of a 3036 bp fragment derived from mouse chromosome 18 containing the first coding exon of *Mro*. This was ligated to a strong splice-acceptor *lac*Z expression cassette (designed to splice onto exon three of *Mro* and act as a reporter), and a neomycin resistance gene under the control of the PGK promoter for selection purposes. Finally, a 3406 bp fragment, which extends downstream from the 3′UTR of *Mro*, was ligated to form the 3′ homology arm. The construct was linearised with *Asc*I and transfected by electroporation into 1×10^7^ ES cells of a line (48/1) derived from 129Ola [15] (a kind gift from Prof. Richard Gardner). After ten days of selection with Geneticin, five hundred resistant colonies were picked and expanded into 96 well plates. Genomic DNA was isolated by digestion with *Proteinase K* (1 mg/ml) in 50 µl of lysis buffer (10 mM Tris pH 7.5, 10 mM EDTA, 10 mM NaCl, 0.5% sarkosyl) followed by ethanol precipitation. Once dried, samples were digested overnight with five units *Spe*I (NEB). Digested, electrophoresed DNA was Southern blotted onto Hybond N+ membrane (Amersham) then hybridised with a 282 bp 5′ external probe (generated by PCR: F, 5′-TGGGAGCCTGACAAGTCCTA-3′; R, 5′-GCAAGCAGGGCAAAATGAAGG-3′) which detects a 9.5 kb fragment derived from the wild-type *Mro* allele and a 7.0 kb fragment from the correctly targeted allele. Probes were labelled using the Megaprime kit (Amersham) and hybridised in ExpressHyb (Clontech) following manufacturers' instructions. Twelve clones containing a correctly targeted allele were identified. These clones were further expanded and following digestion of DNA with *Nde*I, Southern blot analysis was repeated using a 790 bp probe (3′ external probe), which detects a 10.9 kb fragment derived from the wild-type *Mro* allele and a 7.1 kb fragment from the correctly targeted allele. This ensured that correct targeting had occurred at both ends of the integration. Two ES cell clones (*Mro^tm1H^*-C4 and *Mro^tm1H^*-C10), giving the predicted fragment sizes with both external probes and exhibiting a normal 38, XY karyotype, were then chosen for injection into C57BL/6J recipient blastocysts. Embryos were transferred to pseudopregnant CD1 foster mothers. Male and female chimaeric offspring, identified by the presence of agouti coat colour, were then bred to C57BL/6J mice to assay for germ-line transmission. Agouti coloured offspring of these crosses were biopsied and their genotype determined by Southern blotting. Mice carrying the targeted allele were bred onto 129SvEv, C57BL/6J and C3H/HeH backgrounds, and were also inter-crossed to produce homozygote and wild-type littermates for phenotypic analysis. A multiplex PCR assay, which screened for both the presence of the wild-type *Mro* allele (F, 5′-GAACATCCGGCTCTGTCGTC-3′; R, 5′-CCACGAACCAGGAGGTCAAG-3′) and also for the presence of the targeted allele (F, 5′-AGGCTATTCGGCTATGACTG-3′; R, 5′-CGTCAAGAAGGCGATAGAAG-3′), was developed to permit rapid genotyping of mice. This became the sole method of genotyping within the established colony following confirmation that the PCR assay recapitulated results obtained by Southern blotting. A Y chromosome-specific PCR reaction (F, 5-CTCTGAGTACATCCGTGG-3′; R 5′-GCAATCCTGCTGAACTGC-3′) was used to determine the chromosomal sex of individuals within the colony.

### Embryo harvesting, wholemount *in situ* hybridisation and RT-PCR

Timed matings were used to generate embryos at specific stages. Breeding pairs were set up at approximately 3 pm and vaginal plugs were checked the following morning. Noon on the day of the plug was counted as 0.5 dpc. Wholemount *in situ* hybridisation was performed as previously described [7]. The following probes were used for WMISH: *Sox9* [16]; *3β-HSD* (IMAGE EST clone 580043); *Oct4* [17].

### Reverse transcription polymerase chain reaction (RT-PCR)

Embryos generated by timed matings between heterozygotes (+/*Mro^tm1H^*) were dissected at 13.5 dpc and embryonic testes/mesonephros and tail tissue were collected. Samples were snap frozen in a dry ice-ethanol bath. Following completion of PCR genotyping using DNA from the collected tails, five pairs of testes from each of the genotypes detected (*Mro^tm1H^*/*Mro^tm1H^*, +/*Mro^tm1H^*, and +/+) were separately pooled for RNA extraction. Total RNA was extracted using an RNeasy micro-kit (Qiagen), following manufacturer's instructions, including an on-column *DNase* digestion step. First-strand cDNA synthesis was completed using a dedicated cDNA synthesis kit (Roche cat# 1117831), following manufacturers instructions. Three microlitres of the cDNA product was subsequently used as template for RT-PCR amplifications of *Mro* (F, 5′-CAGCGTGCGGTATTCAGCTT-3′; R, 5′-ACAGCAGGATCTCTGGATGGCAGTG-3′) and *Hprt* (F, 5′-AAGGACCTCTCGAAGTGTTG-3′; R, 5′-GACGCAGCAACTGACATTTC-3′). Genomic DNA from 129Ola mice was used as a control of possible genomic DNA contamination.

### Fetal immunohistochemistry: Laminin, 3β-HSD and PECAM detection

Tissues were freshly frozen in OCT compound (BDH/Merck) and cryosections were cut at 10 µm, air-dried and fixed for 15 minutes in ice-cold acetone. Sections were rinsed in PBGT (0.5% gelatine/0.05% Tween 20 (Sigma) in PBS) and then incubated with rat anti-mouse laminin A chain (Chemicon) or rat anti-mouse CD31-PECAM (BD Pharmingen) at a 1∶100 dilution. To detect 3β-HSD sections were incubated with rabbit anti-human 3β-HSD (a gift from Professor Ian Mason) at a 1∶500 dilution in PBGT for 45 minutes at room temperature. Sections were then washed in PBGT and incubated in donkey anti-rat Alexa Fluor 594 (laminin, CD31-Pecam), or donkey anti-rabbit Alexa Fluor 488 (3β-HSD), at a 1∶200 dilution in PBGTN (PBGT+5% Sheep serum obtained from Sigma) in the dark for 45 minutes at room temperature. All Alexa Fluor conjugates were obtained from Invitrogen. After rinsing in PBGT, sections were mounted under coverslips in Vecta Shield (Vecta Laboratories Inc.) and photographed using an Axiophot epifluorescent microscope (Zeiss).

### Fetal immunohistochemistry: AMH and hVASA detetection

Tissues used for AMH and hVASA (also known as DDX4) detection were fixed overnight in 4% paraformaldehyde and embedded in paraffin wax. Sections were then cut at 8 µm thickness and these were de-waxed through a xylene series and rinsed briefly in water. Antigen retrieval was performed using antigen unmasking solution (Vecta Laboratories) following manufacturer's instructions. Sections were pre-blocked in 1% BSA (Sigma), 10% Donkey Serum (Serotec), 0.1% TritonX-100 (Sigma) in PBS at room temperature for 45 minutes. Sections were then incubated in goat anti-human AMH antibody (Santa Cruz) or goat anti-human VASA antibody (R&D Systems) at a 1∶100 dilution at 4°C over night. Samples were washed in PBS containing 0.1% BSA and incubated in Donkey anti-goat Alexa Fluor 594 (AMH) or Donkey anti-goat Alexa Fluor 488 (hVASA) in the dark for one hour at room temperature. After washing, sections were mounted under coverslips in Vecta Shield and photographed as described above.

### DNA microarray analysis

DNA microarrays containing the RNG-MRC 25 K mouse probe set were supplied by the MRC Harwell core microarray facility. Each contained 25,365 spotted oligonucleotide probes of between 50–53 bases in length representing the total number of transcripts predicted for the mouse genome (annotation available at http://www.microarray.fr:8080/mediante/index). Total RNA was extracted from homozygous mutant (*Mro^tm1H^*/*Mro^tm1H^*) embryonic male gonads (n = 20) and an identical number of wild-type (+/+) control gonads at 13.5 dpc. RNA extraction was performed using the RNeasy micro-kit (Qiagen). Approximately 1.0 µg total RNA was used for first-strand cDNA synthesis (Roche cat# 1117831). One fifth of the first-strand reaction was used as template for a global amplification reaction [18]. Amplified cDNA was then fluorescently labelled using the Bioprime labelling kit (Invitrogen). Microarrays were hybridised as previously described [18] and washed in 2× SSC for 5 minutes, 0.1× SSC/ 0.1% SDS for five minutes and 0.1× SSC for 2 minutes, all at room temperature with agitation. After washing, the slides were scanned using a ProScanArray HT (Perkin-Elmer) and data extracted with ImaGene 6.0 (*BioDiscovery*). Normalisation was performed using a 2D Lowess function within the R statistical package (http://www.r-project.org). Data were then imported into GeneSpring (*Silicon Genetics*) for further analysis. The criterion for differential expression was above 2.0-fold change in all replicate hybridisations. These data have been deposited in the ArrayExpress microarray database of the European Bioinformatics Institute (http://www.ebi.ac.uk/arrayexpress/) with accession number E-MEXP-527).

### Fertility assessment

Seven *Mro^tm1H^* homozygous male mice were mated to a total of 25 wild-type females, resulting in the production of 204 pups (95 female, 109 male), with an average of 8.16 pups per litter. Five homozygous mutant females were each mated to wild-type males and all produced litters (25 males and 19 females, average litter size 8.8).

### Adult Pathology, Histology and Immunohistochemistry

A histopathological screen for gross abnormalities was performed on 8-week-old homozygous mice. Two male and two female mice were investigated in a wide (approximately 30 organs and tissue) pathology screen according to a standardized necropsy SOP (http://www.eumorphia.org/EMPReSS/). A further three males and five females were investigated in a narrower histology screen that included brain, adrenal, trachea, lung, diaphragm, aorta, heart, spleen, lymph node (tracheobronchial and mesenteric), esophagus, small and large intestine, liver, gall bladder, exocrine and endocrine pancreas, kidney and ureter. The ovaries and tubular genitalia were also examined in females.

To examine the adult testes of mutant mice in more detail, urogenital systems from wild type and *Mro^tm1H^* homozygous males were removed, fixed in Bouins for 4 to 6 hours, then transferred to 70% ethanol. Testes were then dissected out, weighed and processed and embedded in paraffin wax using standard techniques and an automated processor. Subsequently, sections were cut, mounted onto slides and immunostained for markers of different testicular cell-types, either smooth muscle actin - peritubular myoid cells (Sigma, monoclonal antibody, 1∶5000 dilution), 3β-HSD - Leydig cells (gift from Professor Ian Mason, rabbit polyclonal antibody, 1∶3000), or hVASA - germ cells (Abcam, polyclonal antibody, 1∶500), as detailed previously [19], [20]. Sections were photographed using a Provis microscope (Olympus Optical Co., London, UK) equipped with a Kodak DCS330 camera (Eastman Kodak Co., Rochester, NY). Testis sections from *Mro* homozygotes and wild-type siblings were also immunofluorescently stained for beta-galactosidase (Promega, monoclonal antibody, 1∶400). Following antigen retrieval [19], [20], nonspecific binding sites were blocked by incubating sections in normal goat serum (NGS; Autogen Bioclear UK Ltd.) diluted 1∶4 in PBS containing 5% BSA (Sigma-Aldrich) for 30 minutes, then incubated overnight at 4°C with Beta-galactosidase antibody, diluted 1∶400. Sections were washed three times in PBS and exposed to goat anti-mouse Peroxidase FAB for 30 minutes. Sections were again washed three times in PBS, then underwent tyramide signal enhancement (Perkin-Elmer, NEL741) for 10 minutes, following manufacturer's instructions. Sections were again washed three times in PBS, counterstained with propidium iodide (1∶10000 dilution), mounted, and stored in the dark at 4°C prior to visualisation. Fluorescent images were captured using a Zeiss LSM 510 Meta Axiovert 100 M confocal microscope (Carl Zeiss Ltd.). Appropriate negative controls were included, whereby the primary antibody was replaced by normal goat serum alone, to ensure that any staining observed was specific.

### Ethics

Investigations on mice described here were conducted in accordance with the International Guiding Principles for Biomedical Research Involving Animals as promulgated by the Society for the Study of Reproduction. These experiments were performed under the authority given by UK Home Office Project License PPL 30/1879.

## Results

### Disruption of *Mro* by homologous recombination in ES cells

**Figure 1:**
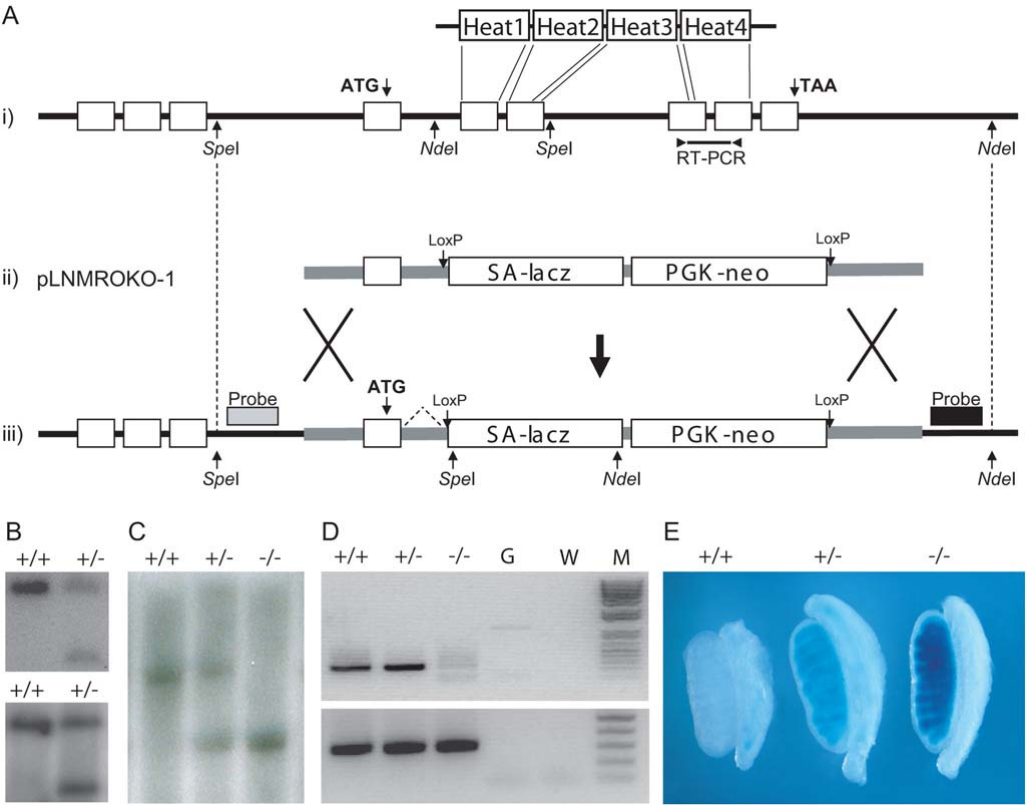
Targeted disruption of the Maestro gene. (A) The mouse Maestro locus (i), targeting vector (ii) and targeted allele (iii). The first coding exon is indicated by the ATG start codon. The final coding exon is indicated by the TAA stop codon. Relationship between exons and HEAT domains in the Mro protein is indicated. The *Nde*I and *Spe*I restriction enzyme sites are shown, as are the primer binding sites for the RT-PCR reaction. The 5′ (shaded box) and 3′ (black box) external probes used for Southern analysis are shown. (B) Southern blot of *Spe*I-digested ES cell DNA samples probed with the 5′ external probe showing wild-type (+/+) and homologous recombinant (+/−) clones. Southern blot of *Nde*I-digested ES cell DNA samples probed with the 3′ external probe showing wild-type (+/+) and homologous recombinant (+/−) clones, confirming that the correct recombination events have occurred at both ends. (C) Southern blot genotyping using the 5′ external probe of three mice derived from intercrossing of offspring of chimaeric mice. Wild-type (+/+), heterozygous (+/−) and homozygous (−/−) samples are shown. (D) RT-PCR analysis of male embryonic gonad (13.5 dpc) RNA samples showing the expression of *Mro* in wild-type (+/+) and heterozygous (+/−) samples but absence of expression in the homozygous (−/−) sample. The housekeeping gene *Hprt* is expressed in all three samples. G, genomic DNA control; W, water control; M, markers. (E) Representative testes from 13.5 dpc wild-type (+/+), heterozygous (+/−) and homozygous (−/−) embryos assayed for Beta-galactosidase activity, showing LacZ is expressed from the correctly targeted *Mro* locus and recapitulates wild-type *Mro* expression [9].

We generated a targeting construct that removed 88% of the coding region of the *Mro* gene (exons 5 to 9) replacing it with an in-frame *lacZ* reporter and a selectable marker. This targeting removes all four HEAT domains of Mro, leaving a single coding exon (exon 4) which splices directly onto the LacZ reporter gene. The design of the targeting construct removes any possibility of generating any of the five splice-variants of *Mro* reported in the Emsembl database (www.ensembl.org/mus), or indeed any novel protein-coding Mro splice-variants (other than the LacZ fusion protein). Mice carrying the targeted mutation (*Mro^tm1H^*), generated from two independent ES cell clones, were identified by Southern blotting and the absence of *Mro* expression in homozygotes was confirmed by reverse transcription polymerase chain reaction (RT-PCR) using RNA from five pairs of testes from each of the genotypes: *Mro^tm1H^*/*Mro^tm1H^*, +/*Mro^tm1H^*, and wild-type littermates. Correct targeting was further confirmed through X-gal staining of 13.5 dpc embryos, which revealed Beta-galactosidase expression recapitulated Mro expression exactly (Fig. 1). These data also confirmed that the first 30 residues of Mro retained in this targeted mouse line were, as expected, expressed as a fusion protein with Beta-galactosidase. Finally, localisation of this fusion protein to the cytoplasm (as opposed to Mro nuclear localisation (9)), strongly suggested that the remaining residues of Mro were extremely unlikely to rescue any potential phenotype. Male and female homozygotes were born in the expected Mendelian ratios and appeared to be morphologically and behaviourally normal by gross inspection. Seven male homozygotes were mated with wild-type females and each sired litters of normal size. Five female homozygotes were also mated with wild-type males and all produced litters with an average size of 8.8.

### Examination of gonad development in male *Mro^tm1H^* homozygotes

**Figure 2:**
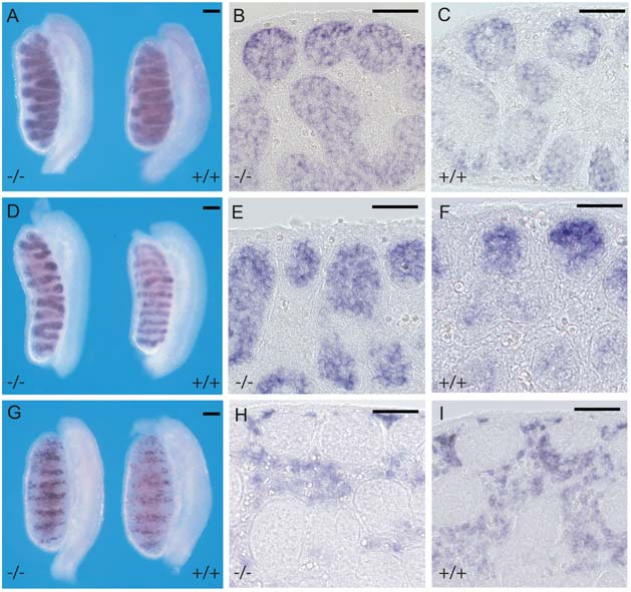
Molecular marker analysis of *Mro^tm1H^* homozygous embryonic male gonads. (A) Wholemount in situ analysis of developing male gonads (13.5 dpc) explanted from wildtype (+/+) and homozygous mutant (−/−) embryos using a probe for the Sertoli cell marker *Sox9*. (B, C) transverse sections through the gonads reveals expression in the testis cords, with signal concentrating at the periphery of the cords. (D) Analysis of expression of the germ cell marker *Oct4*. (E, F) Expression is observed in the testis cords, the signal being restricted to large round germ cells in the centre of the cords. (G) Analysis of expression with the Leydig cell marker *3βHSD*. (H, I) Expression is restricted to cells of the interstitium. No significant differences between wild-type and mutant samples is observed with any of these markers. Scale bars show 50 µM.

**Figure 3:**
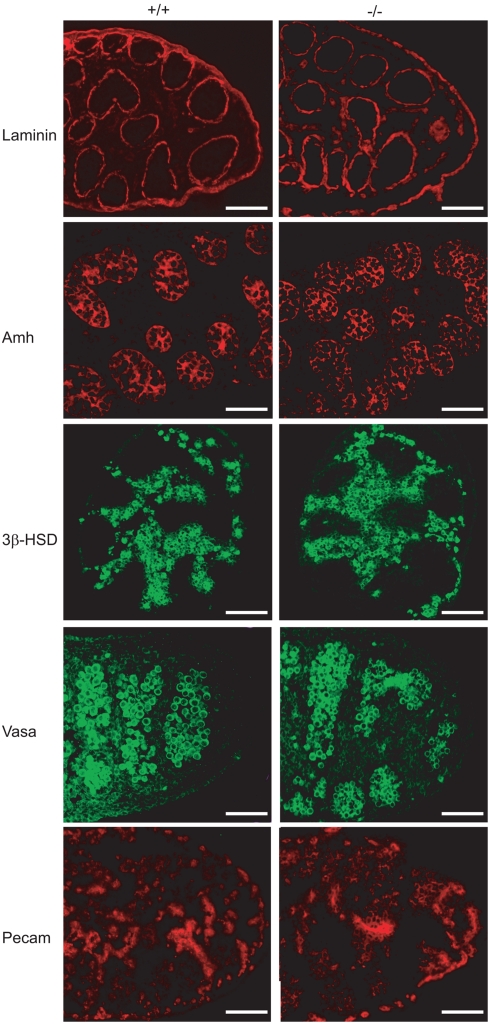
Immunohistochemical analysis of wild-type (+/+) and mutant (−/−) embryonic male gonads. Sections of embryonic gonads (13.5 dpc) are shown after immunostaining with antibodies against the following proteins: Laminin (basement membranes); Amh (Sertoli cells); 3βHSD (Leydig cells); hVASA (germ cells); PECAM (endothelial and germ cells). No significant differences were observed between mutant and wild-type samples with any of the antibodies studied. Scale bars show 50 µM.

In order to determine whether the loss of Maestro had disrupted gonad development, male and female wild-type and homozygous mutant embryos were collected at 12.5 and 13.5 dpc, shortly after sexual differentiation, and dissected gonads were visually inspected for abnormalities. Mutant gonads appeared normal and there were no discrepancies observed between phenotypic (gonadal) sex and genotypic (chromosomal) sex. Embryonic male wild-type and homozygous mutant gonads (13.5 dpc) were then studied in more detail by comparative expression analysis of a number of markers of testicular differentiation, including *Sox9* (Sertoli cell lineage), *3β-HSD* (Leydig cells), and *Oct4* (germ cells). These samples were also sectioned to allow inspection of the cellular specificity of hybridisation. No significant differences between mutant and wild-type gonads were observed (Fig. 2). Antibodies detecting five protein markers of were also used to image the developing male gonads: PECAM, AMH, VASA, 3βHsd and Laminin. Again, no significant differences were observed between homozygous and wild-type gonads (Fig 3).

### Microarray analysis of gonad development

In order to screen for molecular differences between embryonic gonads from homozygotes and wild-type embryos more generally, we used spotted DNA microarrays. Oligonucleotides from 25,365 genes were represented on each array and hybridisations of mutant and wild-type male gonad RNA samples were performed in duplicate. Analysis of these data revealed no significant differences in expression between the samples (see Materials and Methods and data not shown). One oligonucleotide representing Maestro itself, which would have been predicted to hybridise differentially to the two samples, gave a signal that was beneath cut-off levels in both samples. This may represent the low abundance of Maestro, the performance of the oligonucleotide or a combination of these factors. Our previous microarray analysis of Maestro utilised longer spotted cDNA probes [9].

### Histological examination of adult testes in *Mro^tm1H^* homozygotes

**Figure 4:**
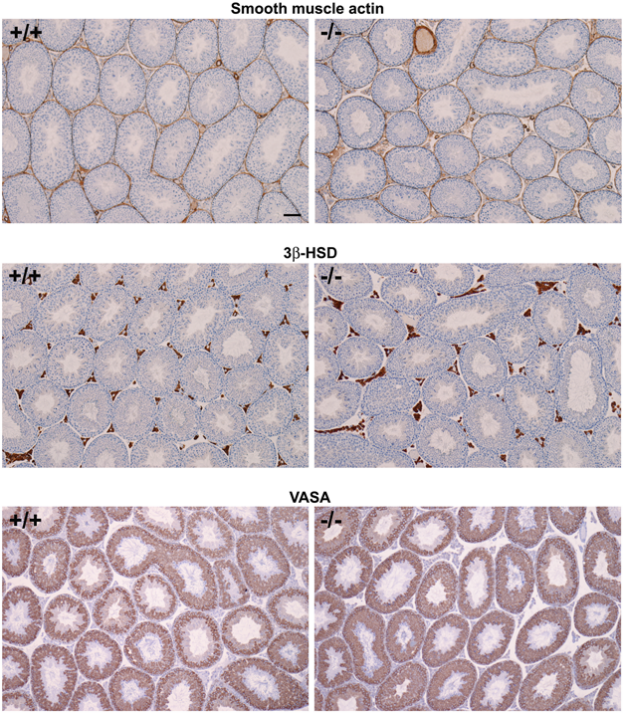
Gross testis morphology in wild-type (+/+) and *Mro^tm1H^* homozygous (−/−) males at age 12 weeks. Sections in the upper two panels were immunostained for smooth muscle actin, which labels peritubular myoid cells and blood vessels, whereas those in the central two panels were immunostained for 3β-HSD, which specifically labels the cytoplasm of Leydig cells. The lower two panels show staining of the germ cell marker, hVASA (DDX4). No significant differences between mutant and wild-type samples were observed. Scale bars show 100 µm.

**Figure 5:**
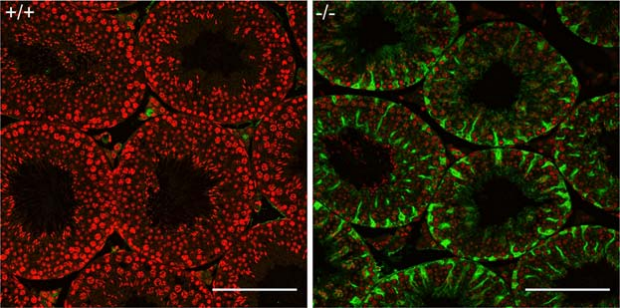
Fluorescent immunohistochemistry against Beta-galactosidase on testis from wild-type (+/+) and *Mro^tm1H^* (−/−) animals (12 weeks). No signal is observed in +/+animals, whereas −/−animals show strong reactivity in Sertoli cell cytoplasm, demonstrating that expression from the *Mro* locus is Sertoli cell-specific in the adult testis (Beta-galactosidase in green; nuclear counterstain propidium iodide in red). Scale bars show 100 µm.

Examination of four 12-week-old *Mro^tm1H^* homozygous males revealed that testis weights in mutant males (100.3±14.2; Mean±SD, N = 4) were unchanged from values for age-matched wild-type males (94.9±26.0). Gross testicular morphology was largely normal in mutant males and most seminiferous tubules exhibited complete spermatogenesis (Fig 4). Immunofluorescent staining of testicular sections from Mro−/− animals demonstrated Beta-galactosidase expression driven by the *Mro* locus is limited to Sertoli cells of the adult testis (Fig 5).

### General histological examinations

Necropsies were performed on twelve 8-week-old homozygous males and females. No gross pathology was found. In addition to the studies of the adult testes, the ovaries and tubular genitalia of homozygous mutant females examined were unremarkable.

## Discussion

Expression profiling using DNA microarrays is an increasingly common method employed to identify candidate sex determining, or sexual differentiation, genes in the mouse. More recently, cell sorting techniques reliant on fluorescently tagged transgenic marker proteins have been employed to purify specific cell types and thereby refine the screen for potential regulators of gonadogenesis [21], [22]. Whilst these and other studies vary in the precise approach taken, they share the common aim of identifying sex- and cell-type specific gene expression in the embryonic gonad as a means of identifying candidates for loci causing disorders of human sexual development. Given that it has been estimated that up to 70% of such cases of gonadal dysgenesis in the human population remain unaccounted for by mutations in known sex determining genes such as *SRY* and *SOX9* [23], such a gene-driven approach to identifying candidates is well motivated. However, very few genes emerging from such screens have made the transition from promising candidate to established player in sexual development. Two recent success stories involving genes initially identified by expression-based screens, the role of prostaglandin D2 in testis determination [24], [25] and the role of *Cyp26B1* as a meiosis inhibiting factor in developing male gonads [26], [27], are relatively rare exceptions to this assessment. Two obstacles to establishing functional roles for the many candidate sexual development genes that exist remain the time and cost of generating mice lacking the relevant gene, and the absence of any reliable *in vitro* assay for gene function during mouse gonadogenesis. However, the generation of mice harbouring null alleles of candidate sexual development genes is perhaps the only reliable way of addressing their function that, at the same time, offers important genetic tools for future experiments.

The expression pattern of the mouse Maestro gene implicates it in some role during sexual development of the male. We have shown that mice lacking Maestro develop normally, are fertile and appear to have no gross morphological abnormalities when housed in conventional conditions. Maestro is highly conserved amongst mammals [9], but no similar gene product has been detected in non-mammalian species by database searches. Moreover, Maestro appears to be a genuine single-copy gene, with no close paralogues existing in mice. Given these observations, the explanation for the absence of any major phenotypic abnormalities in Maestro-deficient mice is unclear. No closely related gene exists that might act redundantly with Maestro, although some unrelated gene product may be able to compensate. We examined gene expression in the developing gonad of Maestro-deficient animals with this explanation in mind, but no significant differences between mutant and wild-type samples were observed. Despite expression of Maestro in extra-gonadal sites, no other morphological abnormalities were observed despite extensive histological examinations. Our observations on mice lacking Maestro have been made with varying degrees of depth on three different genetic backgrounds, namely C3H/HeH, C57BL/6J and 129SvEv. Preliminary studies suggest that there are no significant differences between mutant phenotypes on these backgrounds.

We are currently screening for proteins that physically interact with Maestro during gonad development in the mouse. Once identified, it will be important to determine the phenotypic consequences of removing both Maestro and genes encoding its protein partners. In this way, it may be possible to discern a physiological role for the Maestro gene and identify the relevant molecular pathways in which it acts.

The series of experiments we have undertaken to determine the role of Maestro, whilst inconclusive in their initial aim, have a secondary serendipitous outcome. We have carried out a comprehensive investigation of a locus from which exogenous DNA can be expressed in specific cell lineages of the embryonic and adult testis (with extra-gonadal expression observed in heart, brain, liver and kidney (9)). In addition, by utilizing a LacZ expression cassette, we have demonstrated exactly where to insert such DNA in the *Mro* locus to achieve this expression. Thus whilst we have been unable to determine the function of Mro, the *Mro* locus itself has utility as a site of transgene expression that can be exploited during future investigations into sexual development and Sertoli cell function.
